# Does Mean Platelet Volume Decrease in the presence of Coronary Artery Fistula?

**DOI:** 10.5935/abc.20190088

**Published:** 2019-07

**Authors:** Isa Sincer, Yusuf Çekici, Mehmet Cosgun, Gulali Aktas, Yilmaz Gunes, Emrah Erdal, Asli Kurtar Mansiroglu, Mehmet Inanır

**Affiliations:** 1 Abant Izzet Baysal University Hospital, Bolu - Turkey; 2 Gaziantep Dr. Ersin Arslan Education and Research Hospital, Gaziantep - Turkey

**Keywords:** Arteriovenous Fistula, Coronary Artery Disease, Mean Platelet Volume, Angiography, Endothelium/dysfunction

## Abstract

**Background:**

Coronary artery fistula (CAF) is an abnormal connection that links a coronary artery to a cardiac chamber or another major blood vessel. Several studies have shown the association between mean platelet volume (MPV) and cardiovascular diseases. In the literature, there is no previous study about the association between hematologic parameters and congenital CAF. For this reason, we aimed to investigate the association of MPV with CAF.

**Methods:**

70 patients with normal coronary arteries and 50 with coronary artery fistulas were included. Routine blood and biochemical parameters were measured before the arteriography. Differences between groups for continuous variables were analyzed with t- test or Mann-Whitney test. P values < 0.05 were considered significant. Regression analysis was used to find independent predictors of CAF.

**Results:**

Baseline patient demographics, including age and clinical risk factors, were similar between the groups. Compared to the control group, median (IQR) High-density lipoprotein cholesterol (HDL) levels were significantly higher (p=0.04) and MPV levels were significantly lower in the CAF group (8.84 ± 1.71fL vs. 10.43 ± 1.34, p < 0.001). In the multivariate analysis, only MPV was a significant predictor of CAF (p < 0.001, 95% CI for OR: 0.438 (0.306-0.629). A negative correlation was found between MPV and fistulae in Pearson's correlation test (r: -0.454, p < 0.001). An MPV level of < 9,6 fL showed sensitivity, specificity, positive predictive value and negative predictive value of 80%, 68%, 71% and 78% respectively (AUC = 0.766, 95% CI, 0.678-0.854) for the prediction of CAF.

**Conclusion:**

The present study suggests that MPV may decrease in patients with CAF.

## Introduction

Coronary artery fistula (CAF) is an abnormal connection that links a coronary artery to a cardiac chamber or another major blood vessel.^1^ In the former case, although CAF is generally congenital, the development and dissemination of interventional and surgical techniques over the years, with a higher prevalence of acquired forms, has led to a change in etiology.^2^ CAF is frequently identified incidentally through diagnostic angiography. Very rarely, ischemia may occur due to increased myocardial oxygen demand, and presents with angina or dyspnea on during exertion. The exact incidence of CAF is not certain, since the underdiagnosis rate is high. However, several studies have reported the presence of CAF in 0.13-0.22% of adults undergoing coronary angiography.^3^ Around 75% of incidentally-found CAFs are small and clinically silent. Up to 90% of all CAFs are single and multiple fistulae, present in 10.7-16% of the cases.^4^ Coronary artery fistulae arise more commonly from the right coronary artery (%50-60%) and often drain into the right heart (80%).^1^ A fistula between a coronary artery and a right structure shows a continuous flow from coronary vasculature to the low-pressure right chamber. When the drainage site is located in the left atrium or pulmonary vein, there is an effective left-to-left shunt that determines a volume overload to the left heart only. This volume overload may lead to endothelial dysfunction. In the case of a large-caliber CAF, coronary steal may play a role in myocardial ischemia.^5^ Several studies have shown the association between mean platelet volume (MPV) and cardiovascular diseases.^6,7^

In the literature, there is no previous study about the association between hematological parameters and congenital coronary artery fistulas.

For this reason, we aimed to investigate the association of MPV with CAF.

## Methods

Angiographic data of the patients who underwent coronary angiography (CAG) between 2014 February and 2018 March were retrospectively analyzed. A total of 120 patients were included: 70 with normal coronary arteries and 50 with CAF with no associated critical coronary artery stenosis. Coronary angiography was performed due to ischemic changes in ECG, positive exercise test or myocardial perfusion scintigraphy for ischemia.

Clinical and laboratory findings were obtained by reviewing the patients' files. Hypertension was defined as blood pressure > 140/90 mmHg or receiving antihypertensive medication. Diabetes mellitus was defined as having fasting glucose level > 126 mg/dL or receiving anti-diabetic medication. Presence of total cholesterol > 200 mg/dL or triglycerides > 150 mg/dL was accepted as hyperlipidemia. Patients with a history of acute coronary syndrome in the last 6 months, history of coronary artery stenting or bypass operation, idiopathic dilated or hypertrophic cardiomyopathy, congestive heart failure, moderate to severe renal failure, severe hepatic dysfunction, atrial fibrillation, severe valvular disease, systemic inflammatory diseases (e.g. rheumatoid arthritis, lupus erythematous), malignancy, history of blood transfusion in the last 3 months, recent infection (1 month), leukemia or thrombocytopenia, were excluded. The local institutional board approved the study.

Peripheral venous blood samples were drawn from patients who were admitted for angiography or during regular follow-up checkups. Serum glucose, creatinine, total cholesterol, high-density lipoprotein cholesterol, and low-density lipoprotein cholesterol levels were measured using an automatic biochemical analyzer (Architect C8000, USA). Complete blood count and platelet volume were determined using simultaneous optical and impedance measurements (Cell Dyn 3700; Abbott Diagnostics, Lake Forest, Illinois, USA). Platelet, lymphocyte, monocytes, white blood cell (WBC) and MPV values of each patient were recorded.

Coronary angiographies were performed through the radial or femoral artery. The coronary angiographies were evaluated by three interventional cardiologists who were blinded to the clinical and laboratory data of the patients. The fistula location, drainage site , and the fistula shape were evaluated.

Statistical analyses were carried out using the SPSS 18.0 Statistical Package Program for Windows (SPSS Inc., Chicago, Illinois, USA). The distribution of the variables in the study groups was analyzed by the Kolmogorov-Smirnov test. Normally-distributed variables were compared by *t*-test and expressed as mean ± standard deviation. Variables without normal distribution were compared with the Mann-Whitney U-test and expressed as median (interquartile range). Qualitative variables were expressed as numbers and percentages. The differences between independent groups were assessed by Student's *t*-test for normally-distributed quantitative variables and Mann-Whitney U-test for variables without normal distribution, whereas Chi-square test was used for qualitative variables. Pearson's correlation test was used to assess the correlations of MVP with CAF presence. The univariate analysis was used to disclose the association of variables with CAF. Thereafter, to determine the independent prognostic factors of CAF, the multivariate logistic regression model with the forward-stepwise method was used with variables that were found to be significant in the univariate analysis. A receiver operating curve (ROC) analysis was performed to find MPV sensitivity and specificity, aiming to predict the presence of coronary fistulae. All results were considered statistically significant at the level of p < 0.05.

## Results

Baseline patient demographics, including age and clinical risk factors, were similar between the groups, except that the number of females was significantly lower in the fistula group. Previous medications were also comparable between two groups (Table 1). Compared to the control group, HDL-cholesterol levels were significantly higher in the CAF group. However, serum blood glucose, creatinine and other lipid levels were not significantly different (Table 2).

**Table 1:** General characteristics of the study groups

Baseline characteristics	Fistula (n = 50)	Control (n = 70)	p
Age (mean ±SD) (years))	58 ± 12	55 ± 8	0.123^a^
Male/female	32/18	24/46	0.001^b^
Hypertension (%)	14 (28%)	13 (19%)	0.223 ^b^
Smoking	24 (48%)	35 (50%)	0.829 ^b^
Family history	10 (20%)	12 (17%)	0.115 ^b^
Diabetes mellitus	12 (24%)	9 (13%)	0.113 ^b^
Acetyl salicylic acid	17 (34%)	21(30%)	0.119 ^b^
Statin	15 (30%)	18 (26%)	0.143 ^b^
ACE inhibitor	2 (4%)	3 (4%)	0.938 ^b^
B-blocker	17 (34%)	20 (29%)	0.26 ^b^

ACE: angiotensin-converting enzyme; SD: Standard deviation, data were compared with Student's t-test (^a^) and Chi-square test (^b^).

**Table 2:** Laboratory data of the study cohort

	Fistula (n = 50)	Control (n = 70)	p
Creatinine(mg/dL)	0.80 (0.30)	0.80 (0.22)	0.50^a^
Fasting plasma glucose (mg/dL)	98 (57)	101(17)	0.90 ^a^
LDL-cholesterol (mg/dL)	111 (48)	101(56)	0.47 ^a^
HDL-cholesterol (mg/dL)	42 (16)	44(24)	0.04 ^a^
Triglycerides (mg/dL)	150 (115)	157(107)	0.49 ^a^
Total cholesterol (mg/dL)	190 (54	187(54)	0.56 ^a^
Hematocrit (%)	42 (7)	41(6)	0.45 ^a^
Monocytes (x10^3^ µL)	0.61(0.36)	0.62(0.24)	0.42 ^a^
Hemoglobin (gr/dL)	13.9 ± 1.8	13.3 ± 1.9	0.12 ^b^
MPV(fL)	8.84 ± 1.71	10.43 ± 1.34	< 0.001 ^b^
Platelet count (k/mm^3^)	271 ± 82.72	268 ± 78	0.85 ^b^
Lymphocytes (x10^3^ µL)	2.37 ± 0.751	2.46 ± 1.01	0.60 ^b^
WBC (x10^3^ µL)	8.2 ± 3.1	6.35 ± 2.01	0.18 ^b^

a Data without normal distribution shown as median (interquartile range), acomparison with Mann Whitney U test,

b comparison with t-test. MPV: mean platelet volume; WBC: White blood cells; LDL: low-density lipoprotein cholesterol; HDL: high-density lipoprotein cholesterol.

Although the platelet, lymphocyte, monocytes and WBC counts were not significantly different between the two groups, mean MPV levels were significantly lower in the CAF group (8.84 ± 1.71fL vs. 10.43 ± 1.34, p < 0.001) (Table 2).

**Figure 1:**
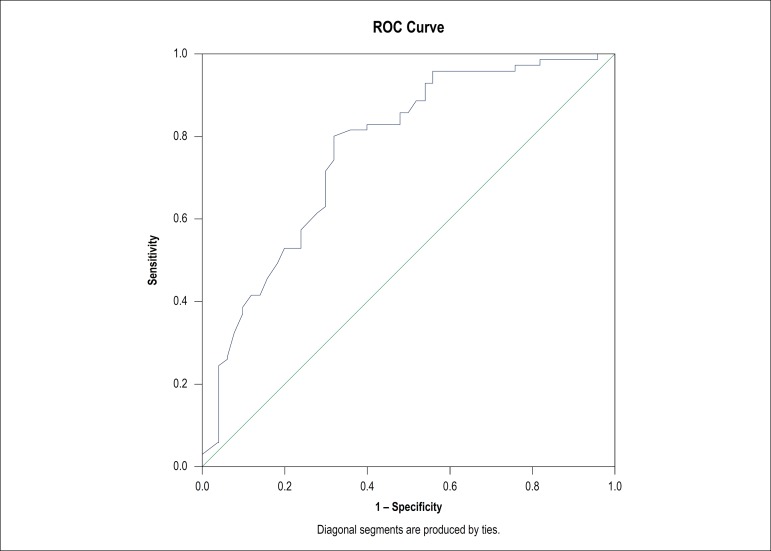
Diagonal segments are produced by ties.

In the multivariate logistic analysis, a forward-stepwise model including MPV, platelet, lymphocyte, monocytes, and WBC counts showed that only MPV was a significant predictor of CAF (p < 0.001, 95% CI for OR: 0.438 (0.306-0.629). A significant negative correlation was found between MPV and fistulae in Pearson's correlation test (r: -0.454, p < 0.001). An MPV level < 9.6 fL had a sensitivity, specificity, positive predictive value and negative predictive value of 80%, 68%, 71% and 78% respectively (AUC = 0.766, 95% CI, 0.678-0.854) for CAF prediction. The overall accuracy of MPV when determining the presence of CAF was 75% (Figure 1).

The right ventricular diameter was slightly increased in the CAF group. Other echocardiographic measurements were similar (Table 3). The angiographic features of the fistula group are summarized in Table 4.

**Table 3:** Echocardiographic findings of the study population

Variables	Fistula	Control	p
Left ventricle end-diastolic diameter(cm)	4.7 ± 0.65	4.6 ± 0.53	0.22
Left ventricle end-systolic diameter(cm)	3.4 ± 0.86	3.3 ± 0.72	0.123
Ejection Fraction (%)	61.4 ± 8.9	63.1 ± 0.76	0.11
Right ventricle end-diastolic diameter(cm)	2.87 ± 0.81	2.46 ± 0.4	0.04
Left atrium diameter(cm)	3.3 ± 0.64	3.4 ± 0.71	0.19

**Table 4:** Angiographic findings of the fistula

Variables	N (%)
**Origin of fistulae**	
RCA	16(32%)
ADA	15(30%)
CX	15(30%)
LMCA	4(8%)
**Drainage site of the fistulae**	
Pulmonary artery	15(%30)
Right ventricle	17(%34)
Right atrium	5(%10)
Left ventricle	13(%26)
**Shape of the fistulae**	
Straight	4(%8)
Single tortuous	21(%42)
Multiple tortuous	25(%50)

RCA: right coronary artery; ADA: anterior descending artery; Cx: circumflex artery; LMCA: main coronary artery.

## Discussion

In this study, we showed that MPV levels were significantly reduced in the CAF group when compared to that with normal coronary arteries. Coronary arterial fistulas are usually asymptomatic. Usual symptoms include dyspnea, angina or fatigue on exertion and, occasionally, arrhythmias. Congestive heart failure may occasionally occur in childhood.^8-11^ In adults, myocardial ischemia may occur due to coronary steal.^12^

In our study, the right ventricular end-diastolic diameters of the fistula group were statistically larger than those in the control group regarding echocardiographic findings. The pathophysiological changes in CAF depend on the pressure difference between the fistula origin and its location and size.^1^ This pressure difference determines the length, the width and tortuosity of the fistula.^1^ A left-to-right shunt is found in over 90% of the cases.^1^ When the fistula drains to the right side of the heart, there is a continuous flow from the fistula due to presence of low pressures in the right chambers, the volume load is increased to the right heart and, consequently, to the pulmonary vascular bed, the left heart chambers. However, when the fistula drains into the left heart chambers, pulmonary blood flow does not increase. Therefore, similar to our findings, right heart chamber dilation is more frequent than left-heart dilation. Especially in the case of a large-caliber CAF, the shifting of blood away from the normal coronary circulation may result in the coronary steal phenomenon and, consequently, in ischemia.^1^

Automatic blood count analyzers calculate MPV in routine assays. It is affected by inflammation and it is accepted as a marker of thrombocyte activation.^13^ A number of diseases, such as metabolic syndrome, myocardial infarction, acute ischemic stroke and diabetes mellitus have been associated with increased MPV.^13,14^ Conversely, lower MPV has been reported in subjects with rheumatoid arthritis, Nasal Polyps and ankylosing spondylitis.^15,16^ Similarly, several studies in the literature reported decreased levels of MPV in irritable bowel disease.^17,18^ It has been proposed that lower and higher MPV values in different conditions are related with high and low-grade inflammation states. Gasparyan et al.^19^ reported that diseases characterized by marked inflammation (e.g. rheumatoid arthritis) are associated with lower MPV values, while low-grade inflammation (e.g. nasal polyps, Behcet's disease) are associated with increased MPV levels.^19^

MPV was found to be higher in most cardiovascular diseases.^13,14^ However, interestingly, MPV in CAF patients was found to be lower than in the control groups. Therefore, we speculate that, unlike acute coronary syndromes, CAF may be associated with a low but continuous inflammatory burden. Unfortunately, this is the first study in the literature that evaluated MPV in CAF. Another author suggested that activated platelets tend to enlarge and cause an elevation in MPV value, and might be utilized in such active inflammatory process, leaving smaller platelets, thus causing a reduction in MPV.^20^ On the other hand, other authors speculate that overproduction of pro-inflammatory cytokines and acute phase reactants can suppress the dimensions of platelets by interfering with the process of megakaryopoiesis in the bone marrow.^13^ HDL-cholesterol was found to have a modulating effect on endothelial dysfunction through antioxidant and anti-inflammatory effects.^21^ Reduced HDL-cholesterol levels in CAF subjects, when compared to controls, may be the underlying cause of endothelial dysfunction in CAF, but prospective studies are still needed to confirm that.

The retrospective cross-sectional design and single-center nature are two important limitations of the present report. Another limitation could be the relatively small study cohort. As far as we know, there are no data about the association of inflammation and CAF. The lack of analysis of inflammatory markers is another important limitation. However, to the best of knowledge, this is the first study that reported an association between CAF and MPV.

## Conclusion

The present study suggests that lower MPV may be associated with CAF. Although an elevated MPV is well established in conditions with higher cardiovascular mortality, this is the first time lower MPV was found in a cardiac disease with low mortality. Therefore, we suggest the use of MPV to determine CAF in patients, considering it is a cost-effective and simple test.
